# A randomized, double-blind, duloxetine-referenced study comparing efficacy and tolerability of 2 fixed doses of vortioxetine in the acute treatment of adults with MDD

**DOI:** 10.1007/s00213-014-3839-0

**Published:** 2015-01-11

**Authors:** Atul R. Mahableshwarkar, Paula L. Jacobsen, Yinzhong Chen, Michael Serenko, Madhukar H. Trivedi

**Affiliations:** 1 CNS Medicine, Clinical Science, CNS Statistics, Pharmacovigilance, Takeda Development Center Americas, 1 Takeda Parkway, Deerfield, IL 60015 USA; 2 Division of Mood Disorders, University of Texas Southwest Medical Center, Dallas, TX USA

**Keywords:** Antidepressant, MDD, Multimodal, ASEX, Treatment-emergent sexual dysfunction

## Abstract

**Rationale:**

Vortioxetine has reduced depressive symptoms in adults with major depressive disorder (MDD) in multiple clinical trials.

**Objectives:**

The aim of this study is to evaluate the efficacy, safety, and tolerability of vortioxetine 15 and 20 mg vs placebo in adults with MDD.

**Methods:**

Patients were randomized 1:1:1:1 to vortioxetine 15 mg, vortioxetine 20 mg, duloxetine 60 mg (active reference), or placebo. The primary efficacy endpoint was mean change in Montgomery–Åsberg Depression Rating Scale (MADRS) total score at week 8 (MMRM). Safety/tolerability assessments included physical examinations, vital signs, laboratory evaluations, electrocardiograms, adverse events (AEs), Columbia–Suicide Severity Rating Scale, Arizona Sexual Experiences Scale, and Discontinuation–Emergent Signs and Symptoms checklist.

**Results:**

Six hundred and fourteen patients were randomized. Mean changes in MADRS scores were −12.83 (±0.834), −14.30 (±0.890), −15.57 (±0.880), and −16.90 (±0.884) for placebo, vortioxetine 15 mg (*P* = .224), vortioxetine 20 mg (*P* = .023), and duloxetine 60 mg (*P* < .001) (*P* vs placebo), respectively. AEs reported by ≥5 % of vortioxetine patients included nausea, headache, diarrhea, dizziness, dry mouth, constipation, vomiting, insomnia, fatigue, and upper respiratory infection. Treatment-emergent sexual dysfunction, suicidal ideation or behavior, and discontinuation symptoms were not significantly different between vortioxetine and placebo.

**Conclusions:**

Vortioxetine 20 mg significantly reduced MADRS total scores after 8 weeks of treatment. Both vortioxetine doses were well tolerated.

**Clinical trial registration:**

ClinicalTrials.gov identifier NCT01153009; www.clinicaltrials.gov/.

## Introduction

Although numerous antidepressants are available for the treatment of major depressive disorder (MDD), only about one third of patients treated with an antidepressant achieve remission in the acute phase (Trivedi et al. 2006). Moreover, even after multiple antidepressant therapies, over 30 % of patients remain symptomatic in the short term (Rush et al. 2006). Vortioxetine is a new antidepressant for the treatment of MDD. The mechanism of action of vortioxetine is thought to be related to a combination of two pharmacological modes of action: direct modulation of receptor activity and inhibition of the 5-HT (serotonin) transporter. In vitro studies indicate that vortioxetine is a 5-HT_3_, 5-HT_7_, and 5-HT_1D_ receptor antagonist, a 5-HT_1B_ receptor partial agonist, a 5-HT_1A_ receptor agonist, and an inhibitor of the 5-HT transporter (Bang-Andersen et al. 2011; Sanchez et al. 2014). The precise contribution of the individual targets to the observed pharmacodynamic profile remains unclear. However, data from serotonergic receptor and transporter occupancy studies, coupled with neuronal firing and microdialysis studies in rats, suggest that these targets interact in a complex fashion leading to modulation of neurotransmission in several systems, including serotonin, norepinephrine, dopamine, histamine, GABA, glutamate, and acetylcholine systems within the rat forebrain (Bang-Andersen et al. 2011; Mørk et al. 2012; Pehrson et al. 2013; Sanchez et al. 2014). This multimodal pharmacological activity is thought to be responsible for the antidepressant effects of vortioxetine.

The efficacy of vortioxetine in reducing depressive symptoms has been demonstrated in patients with MDD in doses from 5 to 20 mg/day (Alvarez et al. 2012; Katona et al. 2012; Henigsberg et al. 2012; Baldwin et al. 2012a; Boulenger et al. 2012; Baldwin et al. 2012b; Jacobsen et al. 2013; Boulenger et al. 2014; Jain et al. 2013; Mahableshwarkar et al. 2013b). Overall, vortioxetine has been well tolerated across all trials. The present phase 3 trial was conducted in the USA and evaluated the efficacy, safety, and tolerability of vortioxetine 15 and 20 mg once daily doses vs placebo in MDD treatment. A duloxetine 60-mg arm was included for trial validation; the study was not powered for direct efficacy comparisons.

## Methods

### Study design

This 8-week, multicenter, randomized, double-blind, placebo-controlled, parallel-group, duloxetine-referenced, phase 3 study was conducted at 58 sites in the USA. Enrollment began in June 2010, and the study was completed in February 2012. The study was approved by individual institutional review boards and conducted in compliance with US Food and Drug Administration (FDA) Code of Federal Regulations Part 56, the International Conference on Harmonisation (ICH) Harmonised Tripartite Guidelines for Good Clinical Practice and the World Medical Association Declaration of Helsinki. After providing signed informed consent, patients entered a 2- to 10-day screening period and, if eligible, were randomized to receive study treatment.

### Rater data monitoring

To control for the possible influence of rater differences, rater assessments were supplemented by having each patient complete a corresponding computerized Montgomery–Åsberg Depression Rating Scale (MADRS) (Montgomery and Asberg 1979) assessment (at screening, baseline, and weeks 1, 2, 4, 6, and 8) and Hamilton Anxiety Rating Scale (HAM-A) (Hamilton 1959) assessment (baseline and weeks 2 and 8) on the same dedicated study laptop computer.

### Patients

Adult men and women (aged 18 to 75 years inclusive) were included if they had a primary diagnosis of recurrent MDD as defined by the *Diagnostic and Statistical Manual of Mental Disorders*, 4th edition text revision with a reported duration of current major depressive episode (MDE) ≥3 months. Patients were required to have a MADRS total score ≥26 at screening and baseline and a Clinical Global Impression–Severity (CGI-S) (Guy 1976) total score ≥4.

Patients were not eligible for study participation if they met any of the following exclusion criteria: treatment with any investigational compound <30 days before screening or five half-lives prior to screening; treatment with vortioxetine in a previous clinical study; a lack of response to previous adequate treatment with duloxetine for any MDE; symptoms considered resistant to two or more antidepressant trials; any concurrent psychiatric disorder other than MDD or prior history of psychiatric disorders such as manic or hypomanic episode, schizophrenia, or substance abuse disorder; significant risk of suicide in the opinion of the investigator or a score of ≥5 on item 10 of the MADRS; or a history of neurological disorders or medically unstable conditions (e.g., renal or hepatic impairment; cardiovascular, pulmonary, or gastrointestinal disorders; pain disorder, chronic fatigue syndrome, fibromyalgia, and obstructive sleep apnea). Additionally, patients were prohibited from receiving formal cognitive or behavioral therapy, systematic psychotherapy, or taking any medication deemed to potentially affect the outcomes of the study. All subjects were required to have a 2-week (or longer depending on drug half-life) washout period for any psychoactive medications prior to screening. After complete description of the study to the subjects, written informed consent was obtained.

### Study treatments

Eligible patients were randomized (1:1:1:1) to receive placebo, vortioxetine 15 mg, vortioxetine 20 mg, or duloxetine 60 mg once daily during the 8-week, double-blind treatment period, using an interactive voice response system. Randomization was stratified by patients’ sexual function status (normal vs abnormal) as determined at baseline by the Arizona Sexual Experiences (ASEX) scale (McGahuey et al. 2000). Abnormal sexual function was defined as having an ASEX total score ≥19 or a score ≥5 on any item, or score ≥4 on any three items (Delgado et al. 2005). Following randomization, doses were up-titrated after the first week of the double-blind period. Patients assigned to receive vortioxetine 15 or 20 mg received a 10-mg dose for the first week of the 8-week study, and those assigned to receive duloxetine 60 mg received a 30-mg dose for the first week.

### Efficacy measures

The MADRS and CGI-S were assessed at screening, baseline, and weeks 1, 2, 4, 6, and 8. HAM-A was assessed at baseline and weeks 1, 2, 4, 6, and 8; the Clinical Global Impressions–Improvement (CGI-I) scale (Guy 1976) at weeks 1, 2, 4, 6, and 8; and the Sheehan Disability Scale (SDS) (Sheehan et al. 1996) at baseline and weeks 6 and 8.

### Safety measures

Adverse events (AEs) were recorded at every study visit after study medication was administered. They were coded by system organ class and preferred term using the *Medical Dictionary for Regulatory Activities* version 11.1. Additional safety measures were assessed as follows: vital signs at weeks 0, 1, 2, 4, 6, and 8; weight, electrocardiogram and laboratory values at weeks 0, 4, and 8; and physical examination findings at week 8. Discontinuation symptoms were evaluated by the Discontinuation–Emergent Signs and Symptoms (DESS) scale during the 2-week discontinuation period. The Columbia–Suicide Severity Rating Scale (C-SSRS) (Posner et al. 2011) was measured at screening, baseline, and weeks 1, 2, 4, 6, and 8. ASEX was assessed at baseline and weeks 1, 2, 4, 6, and 8.

### Statistical analysis

The safety set included all patients who received at least one dose of study medication. The full analysis set comprised all randomized patients who received at least one dose of study drug and had at least one post-baseline value for the primary efficacy assessment. Descriptive statistics and inferential statistics data analysis and tabulations were performed using SAS System, version 9.1.3 (SAS Institute, Inc., Cary, NC) on a Unix platform.

### Statistical methods

The primary efficacy variable—change from baseline MADRS total score at week 8—was analyzed using mixed model for repeated measures (MMRM) analysis of covariance (ANCOVA), with treatment, center, week, treatment-by-week interaction, and baseline MADRS total score-by-week as fixed effects, and a completely unstructured covariance matrix. Based on missing data at random assumption, this analysis was performed using observed case (OC) data only. As sensitivity analysis, the change from baseline in MADRS total score after 8 weeks of treatment was also analyzed using ANCOVA, with treatment and center as fixed factors, and baseline MADRS total score as covariate, using last observation carried forward (LOCF) and OC methods. All statistical tests were two-sided at a significance level of 5 % (except where using corrections for multiplicity), comparing each of the two vortioxetine doses with placebo. Ninety-five percent confidence intervals are presented together with the estimated *P* values.

Changes from baseline in HAM-A total score were analyzed by study visit using both MMRM and ANCOVA (by both LOCF and OC) similar to the methods described above for the primary variable where the baseline score was used as the covariate adjustment in the MMRM and ANCOVA analyses. A similar analysis was performed for CGI-S and CGI-I, where the CGI-S baseline was used as the covariate adjustment in the MMRM and ANCOVA analyses. The same was the case for analysis of SDS total score and subscale change from baseline, where the relevant baseline was used as the covariate adjustment in the MMRM and ANCOVA analyses.

The treatment response, including MADRS response (≥50 % decrease in MADRS), and MADRS remission (MADRS ≤10) were analyzed at all time points by logistic regression adjusting for baseline score and treatment using both LOCF and OC methods.

To control for two-sided type I error, the primary efficacy endpoint and key secondary endpoints were tested for each dose in the following sequential order:

- Change from baseline in MADRS total score at week 8 (MMRM)
- MADRS responders at week 8 (LOCF)
- CGI-I at week 8 (MMRM)
- Change from baseline in MADRS total score at week 8 in patients with baseline HAM-A ≥20 (MMRM)
- MADRS remission at week 8 (LOCF)
- Change from baseline in SDS total score at week 8 (MMRM)

As soon as the test of an endpoint was not significant at a level of .025, the formal testing procedure was stopped. Nominal *P* values with no adjustment for multiplicity were reported for all comparisons between vortioxetine and placebo for subsequent endpoints. The phrase “separation from placebo” was used to describe findings with nominal *P* values <.05.

### Safety variables analysis

All safety assessments including AEs, clinical laboratory evaluations, vital signs, 12-lead electrocardiogram results, and physical examination results were summarized with descriptive statistics, where appropriate. The number of patients with positive reports on the C-SSRS at baseline and during treatment was summarized using descriptive statistics. A report was considered positive if the patient reported any of the following suicidal ideation or behavior (SIB) during treatment: active suicidal ideation with some intent to act without specific plan, active suicidal ideation with specific plan and intent, interrupted/aborted suicide attempt, preparatory acts/behavior, actual attempt, or completed suicide.

The primary ASEX analysis assessed the number of patients who were normal at baseline and developed sexual dysfunction (ASEX total score ≥19, or score ≥5 on any item, or score ≥4 on any three items) anytime during the study period. Change from baseline in ASEX total score and individual items were summarized and analyzed at all time points based on MMRM with treatment, center, week, treatment-by-week interaction, and baseline ASEX total score-by-week as fixed effects.

In patients who completed the 8-week, double-blind period, potential discontinuation symptoms were assessed using the DESS scale, which was administered during a single-blind 2-week period (weeks 9 and 10) following an abrupt discontinuation of vortioxetine treatment. Patients in the duloxetine 60-mg group had their dose tapered to 30 mg for the first week of the discontinuation period.

Comparisons between the different doses of vortioxetine and placebo were performed using an ANCOVA model with treatment and center as factors and the score at week 8 as a covariate. Descriptive statistics were reported for AEs, vital signs, weight, laboratory values, electrocardiogram, and physical examination findings.

## Results

### Patients

**Fig. 1:**
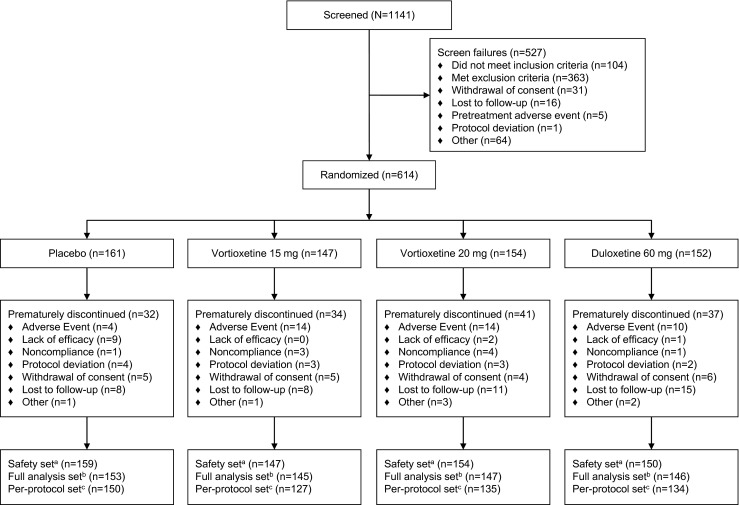
Patient disposition.^*a*^All randomized patients who received ≥1 dose of double-blind study medication; ^*b*^All randomized patients who received ≥1 dose of double-blind study medication and had ≥1 valid post-baseline efficacy assessment; ^*c*^All patients in the full analysis set who had no major protocol violations

Of the 1141 patients screened, 614 (54 %) were randomized (placebo, *n* = 161; vortioxetine 15 mg, *n* = 147; vortioxetine 20 mg, *n* = 154; and duloxetine 60 mg, *n* = 152) (Fig. 1).

### Demographics and baseline characteristics

Demographics and baseline clinical characteristics were balanced across treatment groups (Table 1). In the overall population, 26 % were male, 77 % were White, the mean age was 42.9 years, and the mean body mass index was 31.2 kg/m^2^. The mean duration of the current MDE was 41.2, 38.4, 37.7, and 44.0 weeks in the placebo, vortioxetine 15-mg, vortioxetine 20-mg, and duloxetine 60-mg groups, respectively. Most of the patients (69.4 % overall) had one to three previous MDEs upon entrance into the study, and the majority of patients had been treated for MDEs during the current or previous episode, with 90.4 % overall having received pharmacotherapy.

**Table 1:** Demographics and other baseline characteristics

	Placebo (*n* = 161)	Vortioxetine 15 mg (*n* = 147)	Vortioxetine 20 mg (*n* = 154)	Duloxetine 60 mg (*n* = 152)
Age, mean (±SD) (years)	42.4 (±12.55)	43.1 (±12.28)	42.8 (±12.40)	43.4 (±12.24)
Range	20–73	21–75	19–72	19–72
Female, *n* (%)	116 (72.0)	104 (70.7)	114 (74.0)	119 (78.3)
Race, *n* (%)				
White	122 (75.8)	114 (77.6)	115 (74.7)	119 (78.3)
Black	37 (23.0)	31 (21.1)	36 (23.4)	32 (21.1)
Asian	1 (0.6)	2 (1.4)	3 (1.9)	1(0.7)
Native American/Alaskan native	1 (0.6)	0	0	0
BMI, mean (±SD) (kg/m^2^)	31.1 (±7.88)	31.3 (±7.48)	30.9 (±7.63)	31.5 (±8.45)
MADRS total score, mean (±SD)	31.6 (±4.18)	31.9 (±4.08)	32.0 (±4.36)	32.9 (±4.39)
HAM-A total score, mean (±SD)	17.0 (±5.12)	17.5 (±5.28)	17.8 (±5.42)	18.4 (±5.81)
CGI-S total score, mean (±SD)	4.6 (±0.58)	4.5 (±0.55)	4.5 (±0.60)	4.5 (±0.60)

*BMI* body mass index, *CGI-S* Clinical Global Impression–Severity, *HAM-A* Hamilton Anxiety Rating Scale, *MADRS* Montgomery–Åsberg Depression Rating Scale, *SD* standard deviation

### Efficacy analyses

**Fig. 2:**
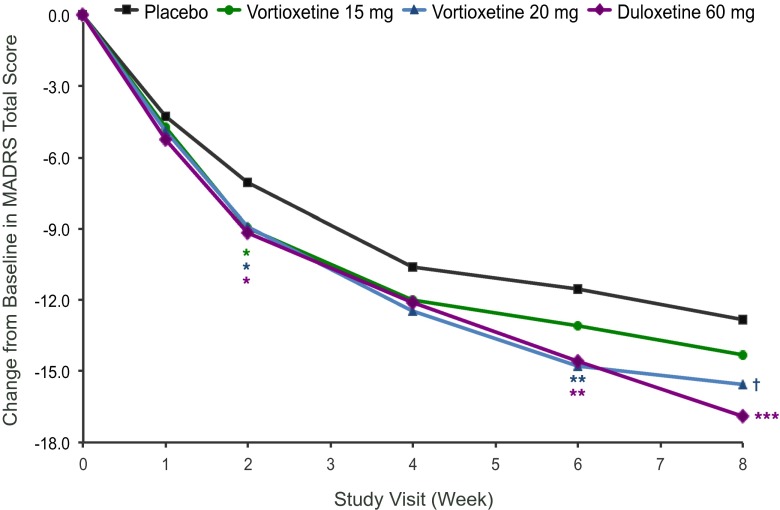
Least-squares change from baseline in Montgomery–Åsberg Depression Rating Scale total score by visit (full analysis set; mixed model for repeated measures). *Nominal *P* < .050; **Nominal *P* < .010; ***Nominal *P* < .001 vs placebo; †*P* < .025

In the primary efficacy analysis, vortioxetine 20 mg was statistically significantly better than placebo (−15.57 ± 0.880 vs −12.83 ± 0.834; *P* = .023) in reducing the MADRS total score at week 8, with a least-squares mean difference from placebo of −2.8 points (Table 2; Fig. 2). Vortioxetine 15 mg was not significantly different from placebo at week 8 (−14.30 ± 0.890 vs –12.83 ± 0.834; *P* = .224). Duloxetine 60 mg separated from placebo (−16.90 ± 0.884 vs −12.83 ± 0.834; *P* < .001) on the primary endpoint, confirming assay sensitivity. In the ANCOVA analyses based on LOCF and OC, neither vortioxetine dose separated from placebo.

**Table 2:** Primary and key secondary endpoints

Variable	Placebo	Vortioxetine 15 mg	Vortioxetine 20 mg	Duloxetine 60 mg
MADRS change at week 8^a^	(*n* = 129) –12.83	(*n* = 113) –14.30	(*n* = 112) –15.57	(*n* = 115) –16.90
Difference from placebo, LS mean (±SE)		−1.48 (±1.214)	−2.75 (±1.206)	−4.07 (±1.214)
*P* value		.224^b^	.023	<.001
95 % CI for difference		(−3.86, 0.91)	(−5.12, −0.38)	(−6.46, −1.69)
MADRS responders at week 8^c^	(*n* = 153) 39.2 %	(*n* = 145) 44.1 %	(*n* = 147) 44.2 %	(*n* = 146) 54.8 %
Difference from placebo (%)		4.9	5.0	15.6
Odds ratio vs placebo		1.249	1.257	1.991
*P* value		.348	.332^b^	.004
95 % CI for odds ratio		(0.786, 1.984)	(0.792, 1.994)	(1.250, 3.171)
CGI-I at week 8	(*n* = 129) 2.65	(*n* = 112) 2.54	(*n* = 111) 2.47	(*n* = 115) 2.31
Difference from placebo, LS mean (±SE)		−0.12 (±0.140)	−0.19 (±0.139)	−0.34 (±0.139)
*P* value		.400	.177	.014
95 % CI for difference		(−0.39, 0.16)	(−0.46, 0.08)	(−0.61, −0.07)
MADRS change at week 8 with baseline HAM-A ≥20	(*n* = 42) –14.27	(*n* = 44) –13.34	(*n* = 34) –14.89	(*n* = 47) –18.31
Difference from placebo, LS mean (±SE)		0.93 (±2.286)	−0.62 (±2.416)	−4.05 (±2.278)
*P* value		.684	.797	.078
95 % CI for difference		(−3.56, 5.45)	(−5.40, 4.15)	(−8.54, 0.45)
MADRS remission at week 8^c^	(*n* = 153) 26.8 %	(*n* = 145) 26.9 %	(*n* = 147) 29.3 %	(*n* = 146) 26.0 %
Difference from placebo (%)		0.1	2.5	−0.8
Odds ratio vs placebo		1.053	1.192	1.098
*P* value		.845	.503	.728
95 % CI for odds ratio		(0.625, 1.775)	(0.713, 1.994)	(0.648, 1.860)
SDS change at week 8^d^	(*n* = 85) –7.68	(*n* = 77) –7.73	(*n* = 77) –8.55	(*n* = 73) –9.66
Difference from placebo, LS mean (±SE)		−0.05 (±1.111)	−0.88 (±1.103)	−1.99 (±1.123)
*P* value		.962	.427	.078
95 % CI for difference		(−2.24, 2.13)	(−3.05, 1.29)	(−4.19, 0.22)

*CGI-I* Clinical Global Impression–Improvement, *HAM-A* Hamilton Anxiety Rating Scale, *LS* least-squares, *MADRS* Montgomery–Åsberg Depression Rating Scale, *SDS* Sheehan Disability Scale, *SE* standard error

^a^Primary efficacy analysis

^b^The testing strategy stopped at this step for all subsequent endpoints with that dose; all subsequent *P* values are nominal

^c^Logistic regression analyses for response and remission (last observation carried forward); values are percentage point differences from placebo

^d^Treatment difference from placebo in mean CGI-I score at week 8

As shown in Table 2, the key secondary efficacy endpoints did not separate from placebo (*P* > .050) with either vortioxetine dose. The mean changes from baseline in SDS total score (MMRM) were numerically larger in the vortioxetine treatment groups compared with placebo; however, neither dose separated from placebo statistically.

### Safety variables

The most common AEs reported in ≥5 % of patients in either of the vortioxetine treatment groups were nausea, headache, diarrhea, dizziness, dry mouth, constipation, vomiting, insomnia, fatigue, nasopharyngitis, and upper respiratory infection (Table 3). Discontinuation due to AEs occurred in 4 (2.5 %) patients in the placebo group, 14 (9.5 %) in the vortioxetine 15-mg group, 14 (9.1 %) in the vortioxetine 20-mg group, and 10 (6.6 %) in the duloxetine 60-mg group (Fig. 1). The most common AE leading to study discontinuation (≥2 % of patients) in the vortioxetine treatment groups was nausea.

**Table 3:** TEAEs experienced by ≥5 % of patients

Patients, *n* (%)	Placebo (*n* = 159)	Vortioxetine 15 mg (*n* = 147)	Vortioxetine 20 mg (*n* = 154)	Duloxetine 60 mg (*n* = 150)
Any TEAE	112 (70.4)	108 (73.5)	125 (81.2)	122 (81.3)
Nausea	18 (11.3)	52 (35.4)	51 (33.1)	55 (36.7)
Dry mouth	16 (10.1)	14 (9.5)	22 (14.3)	26 (17.3)
Headache	21 (13.2)	26 (17.7)	20 (13.0)	28 (18.7)
Dizziness	5 (3.1)	15 (10.3)	20 (13.0)	24 (16.0)
Constipation	10 (6.3)	8 (5.4)	14 (9.1)	18 (12.0)
Vomiting	1 (0.6)	7 (4.8)	13 (8.4)	12 (8.0)
Insomnia	8 (5.0)	5 (3.4)	13 (8.4)	14 (9.3)
Diarrhea	10 (6.3)	22 (15.0)	12 (7.8)	19 (12.7)
Fatigue	4 (2.5)	7 (4.8)	8 (5.2)	17 (11.3)
Nasopharyngitis	10 (6.3)	6 (4.1)	9 (5.8)	5 (5.3)
Upper respiratory tract infection	11 (6.9)	5 (3.4)	8 (5.2)	7 (4.7)

A TEAE was defined as an adverse event with an onset that occurred after receiving study drug and within 30 days after receiving the last dose of study drug

*TEAE* treatment–emergent adverse event

Two serious AEs (a stress fracture and suicidal ideation) occurred in the vortioxetine 15-mg group during the study. The patient with suicidal ideation recovered from the event and continued in the study. There were no deaths among the study participants.

Changes in serum chemistry, hematology, vital signs, and electrocardiogram parameters were distributed evenly across vortioxetine and placebo groups; no discernible patterns of concern were noted for any of the treatment groups.

#### C-SSRS

At the baseline (lifetime) assessment, the incidence of patients with positive C-SSRS reports was similar across the placebo (23.3 %), vortioxetine 15-mg (17.0 %), vortioxetine 20-mg (27.3 %), and duloxetine 60-mg (21.3 %) groups. During the study, one patient in the vortioxetine 15-mg group and one patient in the vortioxetine 20-mg group had active suicidal ideation compared with no patients in the placebo group and two patients in the duloxetine group; no suicidal behavior-related events were reported during the study.

#### ASEX

Approximately one third of patients in each treatment group were without sexual dysfunction at baseline. Of the 58 patients without sexual dysfunction at baseline in the placebo group, 21 patients (36.2 %) developed sexual dysfunction during the study. Of the 45 patients without sexual dysfunction at baseline in the vortioxetine 15-mg group, 16 patients (35.6 %) developed sexual dysfunction during the study; and of the 45 patients without sexual dysfunction at baseline in the vortioxetine 20-mg group, 16 patients (35.6 %) developed sexual dysfunction during the study (Table 4). In the overall study population, there was no statistically significant difference between the vortioxetine treatment groups and placebo regarding the incidence of sexual dysfunction during the study. The vortioxetine 15- and 20-mg groups had a 0.7 % lower rate of sexual dysfunction compared with placebo and a 17.6 % lower rate of sexual dysfunction compared with duloxetine.

**Table 4:** Change in baseline sexual function during study

Variable	Placebo (*n* = 159)	Vortioxetine 15 mg (*n* = 147)	Vortioxetine 20 mg (*n* = 154)	Duloxetine 60 mg (*n* = 150)
Patients without sexual dysfunction at baseline, *n*	58	45	45	47
Without sexual dysfunction during study, *n* (%)	37 (63.8)	29 (64.4)	29 (64.4)	22 (46.8)
With sexual dysfunction during study, *n* (%)	21 (36.2)	16 (35.6)	16 (35.6)	25 (53.2)
Difference in incidence from placebo,%		−0.7	−0.7	17.0
95 % CI for difference^a^		(−19.32, 18.02)	(−19.32, 18.02)	(−1.90, 35.87)
Patients with sexual dysfunction at baseline, *n*	96	98	102	98
Did not worsen during study, *n* (%)	70 (72.9)	80 (81.6)	69 (67.6)	70 (71.4)
Worsened during study, *n* (%)	26 (27.1)	18 (18.4)	33 (32.4)	28 (28.6)
Difference in incidence of worsening vs placebo (%)		−8.7	6.3	1.5
95 % CI for difference^a^		(−20.45, 3.02)	(−6.51, 19.01)	(−11.12, 14.10)

Defined as Arizona Sexual Experiences Scale total score ≥19 or score ≥5 on any item, or score ≥4 on any three items

*CI* confidence interval

^a^Asymptotic 95 % CIs are calculated for proportion difference

When the ASEX scores were analyzed by sex, there were also no differences from placebo for either male or female patients at week 8 in the vortioxetine treatment groups.

#### DESS

Abrupt treatment discontinuation with vortioxetine 15 and 20 mg resulted in no statistically significant differences in DESS total scores compared with placebo at week 9 (placebo, 1.1; vortioxetine 15 mg, 1.8; vortioxetine 20 mg, 1.8) or week 10 (placebo, 1.7; vortioxetine 15 mg, 2.5; vortioxetine 20 mg, 1.6) of the 2-week, single-blind discontinuation period. Following dose-tapering, differences from placebo in DESS total scores in the duloxetine group (week 9, 1.5; week 10, 2.7) were not statistically significant.

## Discussion

Vortioxetine 20-mg treatment was statistically significantly superior to placebo on the primary efficacy analysis and demonstrated continual improvement over the 8-week study. Change from baseline in MADRS total score in the vortioxetine 15-mg group demonstrated improvement over time and was numerically greater than placebo but not statistically significant at week 8. The active reference, duloxetine 60 mg, separated from placebo, confirming assay sensitivity. Neither vortioxetine dose separated from placebo on any of the key secondary efficacy endpoints. Although some improvements in SDS total scores were apparent in the vortioxetine 20-mg and duloxetine 60-mg groups, the differences from placebo did not reach statistical significance. Overall, in the MDD development program, vortioxetine has been studied at doses from 5 to 20 mg. Efficacy has been replicated at the 5-, 10-, and 20-mg doses in adults, and a dedicated elderly study demonstrated efficacy at 5 mg (Alvarez et al. 2012; Katona et al. 2012; Henigsberg et al. 2012; Baldwin et al. 2012a; Baldwin et al. 2012b; Jacobsen et al. 2013; Boulenger et al. 2014). Vortioxetine 20 mg has been shown to be significantly superior to placebo on multiple depression endpoints (MADRS total score, response rate, CGI-I, SDS, and MADRS in patients with high baseline HAM-A) after 8 weeks of treatment in two other studies, one conducted in the USA and the other conducted outside of the USA (Jacobsen et al. 2013; Boulenger et al. 2014). Additionally, there has been a tendency across studies to see increasing efficacy at higher doses (Alvarez et al. 2012; Henigsberg et al. 2012; Baldwin et al. 2012b; Jacobsen et al. 2013; Boulenger et al. 2014). The reasons for failure of the vortioxetine 15-mg dose to separate from placebo in the current study remain unclear as this dose did demonstrate statistically significant improvement over placebo in a previous study conducted outside of the USA (Boulenger et al. 2014). However, another study evaluating vortioxetine 10 and 15 mg conducted in the USA failed (Mahableshwarkar et al. 2013a), so there has been some inconsistency in efficacy results with the lower doses in the USA. This may be due to population differences in the USA that require higher doses (i.e., greater heterogeneity of the population, higher mean BMI) or may relate to methodological issues, including aspects of study design and conduct (i.e., issues with identifying appropriate patients, large multi-site trials, multiple raters) that have been previously proposed as possible reasons for negative clinical trials in MDD (Bridge et al., 2009; Brunoni et al., 2009; Khan et al., 2010; Mundt et al., 2007).

Drugs that act at multiple targets are hypothesized to have a higher potential for side effects. However, vortioxetine was consistently well tolerated across different study populations when measured by various objective scales in addition to spontaneously reported AEs, as was the case in this study. Patients in this trial received the two highest vortioxetine doses studied to date. Even at these doses, AE discontinuation rates were generally low. Nausea was the most common dose-related AE resulting in study withdrawal. Most AEs were of mild to moderate intensity. The prevalence of nausea was transient and highest during the first week of treatment. SIB was prospectively monitored using the C-SSRS scores in all vortioxetine clinical trials, and there was no evidence to suggest that vortioxetine is associated with an increase in SIB in adults with MDD. Abrupt treatment discontinuation of vortioxetine 15 and 20 mg resulted in no statistically significant differences in the incidence of DESS compared with placebo.

Sexual dysfunction, a frequent side effect of drugs with serotonin reuptake inhibitor properties, is a common reason for treatment discontinuation. A meta-analysis of treatment-emergent sexual dysfunction (TESD) reported that TESD incidence ranged from 26 to 80 % depending on the treatment. Atypical antidepressants such as bupropion, mirtazapine, and nefazodone are less likely to cause TESD (Serretti and Chiesa 2009). In this study, the impact of vortioxetine on sexual dysfunction was assessed with ASEX, a validated patient-reported assessment tool (McGahuey et al. 2000) using duloxetine as an active control. As mentioned previously, the primary ASEX analysis assessed the number of patients who were normal at baseline and developed sexual dysfunction during the study. In this study, 32 % of patients had no sexual dysfunction at baseline. Approximately one third (36 %) of these patients in the placebo and vortioxetine 15- and 20-mg groups reported TESD at some point during the trial. In the duloxetine treatment group, approximately one half (53 %) developed TESD. These results are consistent with vortioxetine having a favorable tolerability profile and a low risk of TESD. In sum, vortioxetine 20 mg/day significantly reduced MADRS total score after 8 weeks of treatment compared with placebo. Vortioxetine treatment groups were comparable to placebo in ASEX, C-SSRS, and DESS scores. Overall, the vortioxetine safety and tolerability profile was favorable.
